# Supplementary material for: Metabolomic Profiling Reveals the Anti-Herbivore Mechanisms of Rice (Oryza sativa)
Source: Int J Mol Sci. 2024 May 29;25(11):5946. doi: 10.3390/ijms25115946 (PMC11172427; doi:10.3390/ijms25115946)
Supplement: Supplementary file 1 [file ijms-25-05946-s001.zip › ijms-2991558- supplementary file revised.pdf]

Table S1 Primer sequences for detecting key gene expression levels

Gene name	Primer sequences（5'-3'）	
TUB	qF: CTCCGACTTACAGTTAGAGC	qR: AGTACTGAATCGACAAGCTC
RAT1	qF: ATCCACTGTCACCATTTGAACA	qR: ATCCACTGTCACCATTTGAACA
UTP6	qF: ACGTTCTGTGTTTCAATGGACC	qR: TCATCTCCGCCAATATCAAGAGT
REXO1	qF: GATCGGCCACGCATTAGAGA	qR: GCACGTAGAGATCGGCGTAA
RAN	qF: GCTGGTCAAGAGAAGAAGGCT	qR: TTGAGATCCACACGGGGAGA
CCNB	qF: AGCCCGAAAAATCACAGAACA	qR: GCTCCCGTACAAATGTTGGC
CDC7	qF: CCATAGCCCACACACACAGT	qR: TCGCATCGTATTGAGCCAGA
APC3	qF: AGCGGAAGGACATTTTAATTCGG	qR: TCCGTAAAACCACCACCTCTT
CDK2	qF: GGGTTTACATTGCGGCTTGT	qR: GCATCATCCTTCCCGTAACCT
E2F2	qF: TCCAGAAAAGGCGTGTTTACG	qR: ATATCGCCACCAATCCACCT
RFC3_5	qF: AGTTGTTTTTAGGGAAGGCGG	qR: ATTGGAATGGTGTCCTCACAAA
MCM3	qF: ACAAGCTGAGGAGACCAACG	qR: GCGCATGAATTTGGTGCTCA
POLD2	qF: AACAGTGGTTTTGGTCGGGA	qR: AGGAAAGGTGGTGCATTCAA
MCM4	qF: TTTGTTGGCTGGAGATCCTGG	qR: TGGATCACGACTTACACTTGC
RPS6	qF: GTGGTGCTATTGTTTCTGATGCTA	qR: CGGCTTCAGCAAGATTAGTTTCT

Gene name	Primer sequences（5'-3'）	
RPS16	qF: ACGGAACTTACCACGACGAA	qR: TTCTGGTATCTGGCTCTGGC
RPL10	qF: AGACACAGGAGATAGTTCGGT	qR: TGTGGTCATCTCAAACCCATTCT
RPL21	qF: AGCAAGCACAAGAATCGGGA	qR: ACCTTCACTTCAACAGGCGT
RPL12	qF: AGGAGGAGAACTTCCTGGTC	qR: AGAACGGAAGTGGATGGGTG
OTC	qF: CACAAGTGCTCTCCAATGCG	qR: CATCAACCTCCTTGCCCCTT
desC	qF: GGGCAAGACCATAGACGCTT	qR: TGGGTATTGCTGTTGGCAGA
tdcB	qF: TTGTGTGTGTGATGAGCGGT	qR: GTCACGTTGATGTTGGCGAG
SDHB	qF: GGCTCCTGTGCCATCAATCT	qR: GCGGCTCATCGTTCTTCCTA
ATP20	qF: CGCCAACAAGCCAAACTGAAA	qR: CAGTCCCGACCAGAATAAGCA
SDHC	qF: GCAAGTCATCGGGAAACAGC	qR: AGCAGAGCGTAGCATGTCAG
ATP3	qF: CAGCTAAAGATTCCCCGCCT	qR: GTCAGGTCCCGCCTAATACG
NDUFS3	qF: TGTTTCAATGCATGCTCGGC	qR: GGGAGTACGCTGAATGGAGT
NDUFS8	qF: CCCCACTACTGCTGTCCAAG	qR: TCTCCGTCCAAAAGCAGGTC
ATP2	qF: GCTGACATACGAAGCAAGCG	qR: CTCCAAGGGAAGAACCGGAC
MDH2-1	qF: TCTTGTTCGGTTGTACGCGA	qR: TTGTCTCCTCTCACAGGGCT
SDHA	qF: GTACGGCTACAGCACCAAGT	qR: TGCTTGGAACCTATGCAGGG
MDH2-2	qF: ATCCGAGCTTCCGTTTTCGT	qR: TCGCCATCTTATCACCAGCC
MDH2-3	qF: TTCTGTGCATGGCTTGGTCT	qR: GCCGAAATGTGGATTCGCAG
MDH2-4	qF: CGCTCCATAATCGACGCTCT	qR: ATTTGCGCGATACTGCGTTC
